# Association between High Normal TSH Levels and Obesity in Women with Anti-Thyroid Autoantibodies (ATAs)

**DOI:** 10.3390/jcm11175125

**Published:** 2022-08-31

**Authors:** Fernanda Velluzzi, Silvia Pisanu, Maura Galletta, Michele Fosci, Gianni Secci, Andrea Deledda, Francesco Boi, Rossella Rodia, Giuseppe Fanciulli, Alessandro Palmerio Delitala, Gianmarco Sainas, Andrea Loviselli

**Affiliations:** 1 Endocrinology, and Obesity Unit, Department of Medical Sciences and Public Health, University of Cagliari, 09124 Cagliari, Italy; 2 Hygiene Section, Department of Medical Sciences and Public Health, University of Cagliari, 09042 Cagliari, Italy; 3 Department of Surgical, Medical and Experimental Sciences, University of Sassari-Endocrine Unit, AOU Sassari, 07100 Sassari, Italy

**Keywords:** obesity, overweight, BMI, waist circumference, TSH, anti-thyroid antibodies, thyroid autoimmunity

## Abstract

A positive correlation between Thyroid-Stimulating Hormone (TSH) and Body Mass Index (BMI) has been reported in many studies, but data on this topic remain controversial, especially when TSH values are in the normal range. Moreover, few studies have evaluated the co-existence of thyroid autoimmunity. This study investigated the role of thyroid autoimmunity in the interconnection between TSH, BMI, and waist circumference (WC) in euthyroid patients with overweight or obesity. We enrolled 902 patients (213 males; mean age ± SD: 45 ± 14 years; mean BMI ± SD: 35.8 ± 6.5 kg/m^2^), with normal serum TSH concentration; anti-thyroid autoantibodies (ATAs) were evaluated in 752 patients (186 males). Patients were divided into four BMI classes, based on WHO criteria, and the relationship between BMI, WC, and TSH was evaluated in the whole sample and compared to ATAs positivity, observed in 235 patients (44 males). No significant difference was found between TSH levels in the BMI classes. A statistically significant correlation between TSH and BMI was found only in ATAs-positive females (N = 191, Spearman rho: 0.149; *p*-value: 0.040). However, this finding was not confirmed when considering the WC. Our study shows a positive correlation only between TSH and BMI in obese women with positive ATAs, suggesting that in these patients, the high normal levels of TSH could be attributed to a mild thyroid failure with a possible worsening obesity-related effect, and both need a careful evaluation.

## 1. Introduction

Obesity and overweight are serious health problems. They are major risk factors for non-communicable diseases and have a high prevalence in all parts of the world, including some less-developed regions, especially among middle-aged women [1,2,3]. Subclinical hypothyroidism (SH), which is characterized by normal serum thyroid hormone levels and increased Thyroid-Stimulating Hormone (TSH) values [4,5], is also widespread in the general population [6], especially among middle-aged women [7], and leads to an increased risk of cardiovascular disease [8] and mortality [9]. The association between obesity and SH could determine a further increase in the risk [10]. The assessment of thyroid function in patients with obesity is frequent in clinical practice [11], but the prevalence of altered thyroid function has shown conflicting findings, ranging in particular from 9.7% to 18.9% for overt hypothyroidism and from 9.4% to 20.9% for subclinical hypothyroidism [12]. On this issue, Valdés et al. [13] suggest the use of a wider TSH reference values range for patients with obesity, as the current reference values would triple the prevalence of hypothyroidism among these patients. Likewise, high normal serum TSH levels are associated with higher BMI values in the general Danish population [14], although the findings on this association remain controversial. In fact, this first observation was confirmed by both population-based [15,16,17,18,19,20] and clinical studies [21,22,23,24,25,26,27] among individuals with obesity, but it was not confirmed by several other population-based [28,29,30] and clinical studies [31,32,33,34,35,36]. Moreover, in patients with obesity, the mean serum TSH values have also shown contrasting results: a meta-analysis by Fontenelle et al. [37] reported mean TSH values higher in patients with obesity than in normal-weight individuals in eight studies, but the values were superimposable in another three studies. Furthermore, Knudsen et al., who evaluated a Danish population ten years after they were initially evaluated in 2005 [14], observed that the changes in TSH levels appeared to be positively associated with weight changes [38]. More recently, several studies have been conducted on obese patients treated with bariatric surgery. Juiz Valina et al. evaluated TSH changes at 1, 3, 6, 12, 18, 24, 30, 36, and 42 months after bariatric therapy in 129 obese euthyroid patients and found a significant decrease in TSH values, especially between 6 and 12 months (*p* < 0.001), of approximately 0.034 units per month of follow-up [39]. In a subsequent study conducted selectively on patients with morbid obesity, the same authors observed a central resistance to thyroid hormones preoperatively, which markedly reduced postoperatively [40]. In a review focused on the effects of bariatric surgery on the alterations of thyroid function in obesity, Cordido et al. suggested that the eventual mild hyperthyrotropinemia may be the consequence of a compensatory response and should be considered non-pathological in the absence of other evidence of reduced thyroid function [41]. It is important to emphasize that highly precise identification of thyroid dysfunction in patients with obesity is not negligible since even a mild thyroid failure or ATAs positivity could increase the risk of cardiovascular diseases [42] or psychopathological disorders [43,44] and make weight loss more difficult [45]. On the other hand, long-term treatment with thyroid hormones in euthyroid patients with obesity does not significantly improve weight loss and may cause adverse effects [46]. In the general adult population, the majority of cases of subclinical and overt hypothyroidism are caused by autoimmune thyroid disease (AITD) [4]. Both obesity [47,48,49,50] and autoimmune thyroid disease [51,52,53] are characterized by low degrees of inflammation, which could favor the onset of one disease on top of the other [54,55,56,57,58]. In a systematic review and meta-analysis, Song et al. confirmed the previously reported link between obesity and thyroid autoimmunity, showing a significant association between obesity and autoimmune hypothyroidism and thyroid peroxidase autoantibodies (TPOAb) [59]. Moreover, it has also been reported that high normal serum TSH values are associated with all-cause mortality and non-fatal cardiovascular events in middle-aged and older overweight women. The authors suggest that this association is probably mediated by the pro-inflammatory state and autoimmune activation affecting the circulating TSH, particularly in the female sex [60]. Although autoimmunity may partially influence the relationship between TSH and BMI in humans [54,61], only few studies, and with conflicting results, have investigated the existence of thyroid autoimmunity in patients with obesity. In this regard, since the thyroid ultrasound scan in patients with obesity shows a hypoechoic pattern that could not be attributed to AITD, Rotondi et al. suggested [62] that ultrasound is not a diagnostic tool in these patients, unlike in the general population [36,63], and only the evaluation of antithyroid autoantibodies (ATAs) would be diagnostic for autoimmune thyroid disease (AITD) in obesity. It is remarkable that most of the studies investigating the relationship between TSH serum levels and obesity reported low numbers of ATAs positive patients, thus limiting the statistical power. Therefore, it is reasonable to assume that the contrasting results concerning the relationship between high normal TSH serum concentrations and obesity may partly depend on the different prevalence rates of underlying autoimmune thyroid diseases in the different studies. Moreover, these studies did not consider the TSH-BMI relationship on the basis of ATAs positivity [33,34,36,64,65]. Recently, in the Tehran Thyroid Study, Mahdavi et al. found an increased prevalence of TPOAb in a wide number of patients with obesity, explaining this association as a possible result of the obesity-related inflammatory and immune alterations in the thyroid gland of these patients [66]. The island of Sardinia presents a high prevalence of several autoimmune diseases [67,68,69,70,71,72,73], as well as a growing prevalence of obesity [74]. For these reasons, a study on Sardinian patients with obesity could reach a sufficient statistical strength to highlight any correlation between thyroid autoimmunity, anthropometric data, and high normal TSH levels. Based on this background, this study aimed to elucidate the role of thyroid autoimmunity in the relationship between thyroid and obesity in Sardinian euthyroid patients affected by overweight or obesity.

## 2. Materials and Methods

### 2.1. Experimental Sample Size

The study sample was retrospectively selected from a population of 1393 outpatients with overweight or obesity consecutively referred to the Obesity Unit of the University Hospital of Cagliari during the years 2015–2018.

Exclusion criteria were the following:

(a) Overt or subclinical hyper/hypothyroidism and/or current or previous specific treatment for thyroid diseases (medical, surgical, radiometabolic) (236 patients);

Hyperthyroidism and hypothyroidism were diagnosed according to the current guidelines. Specifically, overt hyperthyroidism was defined by high serum fT4 or fT3 concentration and undetectable serum TSH concentration, while overt hypothyroidism was defined as low serum fT4 concentration, with elevated serum TSH concentration; subclinical hyperthyroidism was defined as serum TSH concentration below the lower limit of the reference range, with fT4 and fT3 concentration within their reference ranges, while subclinical hypothyroidism was defined as serum TSH concentration above the upper limit of the reference range, with fT4 concentration within its reference range [75,76,77].

(b) History of intake of medications potentially affecting TSH levels (32 patients);
(c) Diabetes mellitus (223 patients).

The study subjects were 902 euthyroid patients (213 males). ATAs were evaluated in 752 of them (186 males).

### 2.2. Anthropometric Data

The anthropometric evaluation was performed by an expert nutritionist as follows:

(a) Height (expressed in cm), measured without shoes, with the participant’s head being in the “Frankfort plane”, by means of a stadiometer (SECA);
(b) Body weight (expressed in kg), measured by wearing light clothes, by means of a mechanical scale (SECA 700);
(c) BMI (Body Mass Index, expressed in kg/m^2^), calculated by the ratio of weight in kilograms and height in meters squared;
(d) Waist circumference (WC) (expressed in cm), measured using recommendations of the Airlie conference (according to WHO criteria), considering as cut-off the International Diabetes Federation (IDF) values (94 cm for men and 80 cm for women) [78].

### 2.3. Blood Sampling and Analytes Measurements

Fasting venous blood samples were taken between 08:00 and 09:00 a.m. from the antecubital vein of the arm.

(a) TSH was analyzed via an ultra-sensitive automatic chemiluminescence method (Ortho-Clinical Diagnostic SpA, Milan, Italy). The sensitivity was 0.004 µUI/L, and normal values were 0.4–4.1 µUI/L.
(b) ATAs (thyroid peroxidase autoantibodies, TPOAb) were measured with a chemiluminescent method (Immulite 2000’s; Diagnostic Products Corporation, Los Angeles, CA, USA; Distributor Medical Systems Corporation, Genoa, Italy), and normal range values were 10–35 IU/mL.

### 2.4. Ethical Committee

All procedures performed in studies involving human participants were in accordance with the ethical standards of the institutional and/or national research committee and with the 1964 Helsinki declaration and its later amendments or comparable ethical standards (AOU Cagliari, Prot.PG/2020/10920).

### 2.5. Statistical Analysis

The results for quantitative variables were expressed as mean value and standard deviation or median and range, based on the distribution of the data. The Kolmogorov–Smirnov (K-S) test showed a non-parametric distribution for TSH serum levels and a parametric distribution for both BMI and WC values. In this sense, TSH values were reported as median (range), and both BMI and WC values were reported as mean ± standard deviation (M ± SD).

Data were analyzed by using a non-parametric Kruskal-Wallis test for unpaired data, and the relationship between the quantitative variables was evaluated using the Spearman correlation test. For all the tests, a *p*-value (*p*) < 0.05 was considered as the limit of significance. All analyses were performed with SPSS version 25.0.

## 3. Results

All patients were aged between 18 and 79 years (M ± SD: 45.0 ± 14.2 years).

Based on BMI values, according to WHO criteria [3], 154 patients (24 males) with an average age of 40.3 years (SD ±13.7) were categorized as being overweight, 290 (70 males) as class I obesity (average age of 45.1 ± 14.3 years), 259 (58 males) as class II obesity (average age of 46.4 ± 14.3 years), and 199 (61 males) as class III obesity (average age of 46.7 ± 13.6). The mean BMI value of the whole sample was 35.8 ± 6.5 kg/m^2^.

In addition, the majority of patients presented WC values above the IDF cut-off: in fact, only seven patients (0.8% of the whole sample, one male) were within the established limits.

Table 1 shows the median values and range of TSH serum concentrations by BMI class: TSH levels were comparable between the four BMI groups (*p =* 0.302).

The patients were then grouped according to the ATAs’ positivity or negativity: positivity was found in 236 (26%) patients (44 males) with an average age of 44.6 ± 14.1 years. Negativity was found in 516 (57%) patients (142 males) with an average age of 44.2 ± 14.2 years. As shown in Table 2, the statistical comparison did not show any difference in TSH, BMI, or WC values between the two groups.

Dividing patients by sex, a slight increase in TSH, BMI, and WC values, although not significant, was observed only in ATAs-positive female patients (N = 191), as shown in Table 3.

Next, we considered the relationship between TSH values and BMI or WC values in terms of the correlation between TSH and the two anthropometric variables in the whole sample, stratified by sex and based on ATAs positivity.

**Figure 1:**
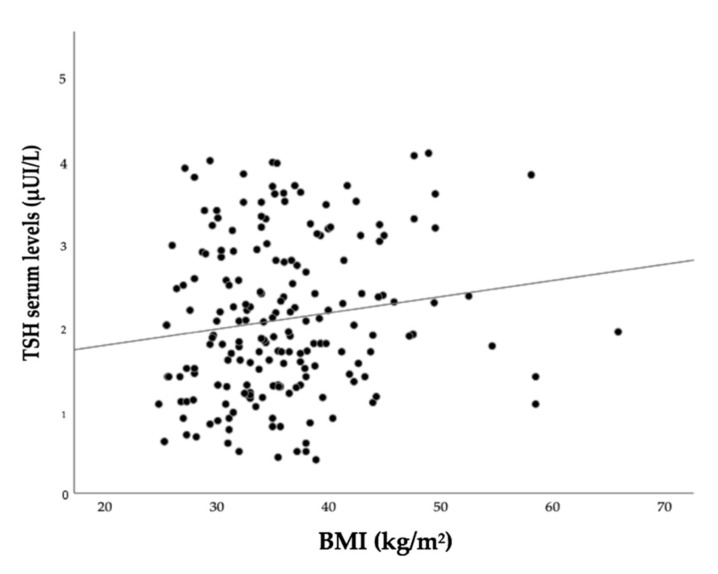
Spearman’s correlation between TSH and BMI in ATAs-positive female patients.

Regarding the BMI, no significant results were obtained in the total sample and in the sample stratified by sex (in total sample: Spearman rho: −0.014; *p*: 0.682; in females: Spearman rho: 0.013; *p*: 0.730; in males: Spearman rho: −0.030; *p*: 0.666). Similarly, no significant results were found when considering only the ATAs-positive patients (N = 236: Spearman rho: −0.099; *p*: 0.130). However, when restricting the analysis to ATAs-positive females (N = 191), a positive correlation between TSH and BMI was observed (Spearman rho: 0.149; *p*: 0.040), as shown in Figure 1.

When considering the WC, no correlation with TSH was found across the whole sample (Spearman rho: −0.013; *p*: 0.703) and stratified by sex (in females: Spearman rho: 0.040; *p*: 0.309; in males: Spearman rho: −0.065; *p*: 0.358). Concerning ATAs status, the correlation was not significant in ATAs-positive patients of both sexes (N = 236, Spearman rho: 0.056; *p*: 0.401), and, in contrast with the findings on BMI, the correlation between WC and TSH was not significant when restricting the analysis to ATAs-positive females (N = 191, Spearman rho: 0.131; *p*: 0.075).

## 4. Discussion

In this study, we found a significant correlation between TSH within the reference range and BMI in ATAs (TPOAb)-positive female patients, despite the range restriction of TSH values possibly decreasing the Spearman’s rho value, which is reported to be lower when the variability between the observed values is small [79]. Therefore, the weakness of the correlation between TSH and BMI observed in our study in ATAs-positive euthyroid female patients could be explained by the restricted range of TSH values in our study population, as suggested by Bland, Altman et al. [80]. The positive association between TSH and BMI in subjects with thyroid autoimmunity is partially supported by the observations derived from population studies, in which the inclusion of ATAs-positive subjects shifted the Gaussian distribution of mean TSH values to the right [81,82]. Moreover, the higher prevalence of thyroid autoimmunity among women is widely reported [83]. In addition, our results are supported by some epidemiological surveys. Kitahara et al. [65] observed a positive correlation between serum TSH concentrations and BMI values among 3114 American adults, but when the 325 ATAs-positive patients were excluded, this result was not confirmed. Chen et al. [42] observed a significantly higher BMI mean value in ATAs-positive women compared with ATAs-negative women in 9082 adult subjects with normal TSH serum concentrations. Furthermore, this study showed that euthyroid AITD patients might have an increased cardiometabolic risk with respect to that associated with obesity per se. Additionally, a clinical study performed in a Spanish endocrine clinic by Diez et al. [64] found a positive correlation between serum TSH levels and BMI in 412 euthyroid obese patients, but this result was not confirmed when the 46 ATAs-positive patients were excluded. This finding is in line with our hypothesis, even if it is surprising that such a low number of ATAs-positive patients (10%), reported by Diez et al., was able to affect the relationship between TSH and BMI. Unlike the BMI, in our study, the waist circumference, a good marker of metabolic risk [84], was not significantly correlated with TSH values, even in ATAs-positive females, although a trend was observed. On this issue, our findings are only partially in line with the results of De Pergola et al. in euthyroid obese female patients [32] and with data reported in some studies on the general population [85,86], in which a correlation between WC and thyroid function was found exclusively in female subjects. However, in these studies, thyroid autoimmunity (ATAs) was not evaluated [32,85] or not considered relevant [86,87].

On the other hand, other studies on Asiatic people did not find any correlation between WC and TSH [88,89].

The main results of the studies mentioned above are summarized in Table 4.

It should be pointed out that in our study, the great majority of patients showed WC values above the cut-off, and the scarcity of values within the reference limits may have affected these results. Our findings could be explained by the hypothesis that obesity itself can play a role in autoimmunity [55,58,90,91], in particular through the action of leptin [92], and that high normal values of TSH may be linked to pro-inflammatory status and autoimmune activation [93]. Despite this possible and not-yet-established causal link, only a few and conflicting studies have investigated the causal role of thyroid autoimmunity on the high normal serum TSH concentrations in patients with obesity [33,35]. In a recent population-based study on more than 12,000 Chinese adults, overweight and obesity were associated with hyperthyrotropinemia exclusively in the presence of thyroid autoimmunity. Based on this result, the authors hypothesized that obesity could worsen the role of autoimmunity on hyperthyrotropinemia [94]. Our results are somewhat consistent with those of the aforementioned study, namely, the detection of higher, although within the normal range, TSH levels in ATAs-positive females. In addition, as expected by our previous studies on a Sardinian population [68,70], we found a higher prevalence (26%) of ATAs positivity in our series compared to the general European population [95,96]. This finding could be considered an important tool in the clinical evaluation of patients with overweight or obesity. In fact, even when TSH values are within the normal range, ATAs determination may be useful to identify patients suffering from AITD. Moreover, in these patients, the possible aggravating role of obesity in the pathogenesis of AITD requires particularly accurate treatment and monitoring of the weight excess. On the other hand, in ATAs-negative patients, the weight reduction could be sufficient to bring TSH values into the optimum range. However, a limitation of the cross-sectional studies is the inability to establish the potentially worsening effect of obesity on the development of thyroid dysfunction in ATAs-positive patients. In fact, only an observational study comparing a sample of obese patients with a sample of normal-weight subjects, both with ATAs positivity, could establish the role of obesity in worsening the progression toward thyroid failure. In the general population, the risk of developing hypothyroidism in women is 4.3% per year in those with raised TSH serum levels and ATAs positivity, 2.6% per year in those with raised TSH alone, and 2.1% per year in those with ATAs positivity alone, but the “weight of the weight” has not been considered [97]. Moreover, a further limitation is the exclusiveness and particularity of the ethnic group we studied, as it is not certain that the influence of autoimmunity is so decisive in other populations. Another possible limitation of our study could be the lack of measurement of free thyroid hormone levels. Nonetheless, only central hypothyroidism, which is rather uncommon, might be missed with a screening of the thyroid function based on TSH evaluation [95]. In addition, the behavior of serum thyroid hormone concentrations has shown a considerable variability in patients with obesity as a result of changes in nutritional habits [98], and in obese patients undergoing bariatric surgery, with the exception of patients affected by AITD [99].

## 5. Conclusions

In conclusion, this study suggests that in obese women with ATAs positivity, the high normal levels of TSH could be attributed to a mild thyroid failure, with a possible worsening obesity-related effect, while in all other cases, obesity per se could play a causal role.

## Figures and Tables

**Table 1:** Median values and range of TSH serum concentrations by BMI class.

Class (BMI)	Number	TSH (μUI/L)
Overweight (25.0–29.9)	154	1.9 (0.4–4.0)
Class I Obesity (30.0–34.9)	290	1.9 (0.5–4.1)
Class II Obesity (35.0–39.9)	259	1.8 (0.4–4.1)
Class III Obesity (≥40)	199	1.9 (0.5–4.1)

Abbreviations: BMI = Body Mass Index (kg/m^2^), TSH = Thyroid-Stimulating Hormone.

**Table 2:** TSH serum levels, BMI, and WC values according to ATAs status in the whole sample.

	ATAs+ Patients (N = 236)		ATAs- Patients (N = 516)		
Variable	Median (Range)	M ± SD	Median (Range)	M ± SD	*p*-Value
TSH (μUI/L)	1.85 (0.7–3.7)		1.86 (0.7–3.5)		0.504
BMI (kg/m^2^)		36.0 ± 6.8		35.8 ± 6.4	0.749
WC (cm)		112.4 ± 14		112.2 ± 14.2	0.907

Abbreviations: ATAs = Anti-Thyroid Autoantibodies (thyroid peroxidase autoantibodies), BMI = Body Mass Index, M = Mean, SD = Standard Deviation, TSH = Thyroid-Stimulating Hormone, WC = Waist Circumference.

**Table 3:** TSH serum levels, BMI, and WC values according to ATAs’ status in female patients.

	ATAs+ Patients (N = 191)		ATAs- Patients (N = 375)		
Variable	Median (Range)	M ± SD	Median (Range)	M ± SD	*p*-Value
TSH (μUI/L)	1.91 (0.7–3.7)		1.90 (0.8–3.6)		0.412
BMI (kg/m^2^)		36.0 ± 6.9		35.1 ± 6.1	0.241
WC (cm)		111 ± 14.1		109.5 ± 13.2	0.194

Abbreviations: ATAs = Anti-Thyroid Autoantibodies (thyroid peroxidase autoantibodies), BMI = Body Mass Index, M = Mean, SD= Standard Deviation, TSH = Thyroid-Stimulating Hormone, WC = Waist Circumference.

**Table 4:** Association between anthropometric measures and TSH in euthyroid adults.

Author	People Assessed	Results
Kitahara [66]—USA	3.114 euthyroid adults	TSH positively associated with BMI only when ATAs-positive patients were considered
Chen [42]—Japan	9.082 euthyroid adults	TSH positively associated with BMI in ATAs-positive women
Diez [65]—Spain	412 euthyroid obese adults	TSH positively associated with BMI only when ATAs-positive patients were considered
De Pergola [32]–Italy	201 euthyroid women	TSH positively associated with WC
Diniz [87]—Brazil	11.224 euthyroid adults	TSH associated with BMI and WC only in women
Delitala [86]—Italy	4.733 euthyroid adults	TSH not associated with anthropometric measures
Liu [89]—China	13.505 euthyroid adults	TSH not associated with WC
Sakurai [90]—Japan	2.037 euthyroid adults	TSH not associated with WC

Abbreviations: TSH = Thyroid-Stimulating Hormone, BMI = Body Mass Index (g/m^2^), ATAs = Anti-Thyroid Autoantibodies, WC = Waist Circumference.

## Data Availability

The datasets used and/or analysed during the current study will be made available by the authors on reasonable request.
